# Evaluation of The Metabolic Syndrome Criteria And Body Composition in Ambulatory Children with Epilepsy UsingSodium Valproate and Carbamazepine In Southern Iran: A Case-Control Study

**Published:** 2020

**Authors:** Soroor INALOO, Forough SAKI, Mohammad PAKTINAT, Pegah KATIBEH, Hamid NEMATI, Gholamhossein RANJBAR OMRANI

**Affiliations:** 1 Neonatal Research Center, Shiraz University of Medical Sciences, Shiraz, Iran; 2 Endocrinology and Metabolism Research Center, Shiraz University of Medical Sciences, Shiraz, Iran.; 3 Pediatric neurology ward, Shiraz Neuroscience Research Center, Shiraz University of Medical Sciences, Shiraz, Iran.

**Keywords:** Body Composition, Children, Epilepsy, Metabolic Syndrome

## Abstract

**Objectives:**

Previous studies in adults with epilepsy revealed a higher prevalence of metabolic syndrome, resulting in cerebrovascular and cardiovascular events. However, there is insufficient data about body composition and metabolic syndrome in children, especially in the Middle Eastern region. We aimed to investigate metabolic syndrome criteria and body composition in ambulatory children with Epilepsy in Southern Iran.

**Material & Methods:**

Seventy seven epileptic children with an average age of 11.4 ± 3.2 years and their age-gender-matched controls were included in this study. Anthropometric data, lipid profile, blood glucose, and blood pressure were checked. Body composition was also evaluated by Hologic system dual-energy X-ray absorptiometry.

**Results:**

The prevalence of metabolic syndrome as well as the fat mass index in patients were higher than the controls, and p values are 0.032 and 0.012, respectively. Moreover, the lean mass with Bone Mineral Content (BMC) index was detected lower than the controls (P= 0.017).

Regarding drugs consumption, serum triglyceride and the blood pressure in patients who receiving carbamazepine was higher than the control individuals with P = 0.019, Beta = 0.379 and P = 0.016, Beta = -0.26, respectively. Fat mass index was also higher in patients using sodium valproate (P = 0.031, Beta = 0.238).

**Conclusion:**

Our study revealed that children with epilepsy are more prone to metabolic syndrome and higher body fat mass. Therefore, early diagnosis and prevention of metabolic syndrome criteria in patients with epilepsy, With performing regular exercise and having a healthy diet should be encouraged in these children.

## Introduction

Cardiovascular diseases are one of the most important leading causes of morbidity and mortality worldwide (1). Metabolic syndrome (MS) is defined as a group of metabolic risk factors, including glucose intolerance, dyslipidemia, hypertension, and central obesity as the main risk factors for cerebrovascular and cardiovascular events (2). Urbanization, fast food, and sedentary lifestyle have led to an increase in the prevalence of obesity and metabolic disturbances in developing countries children (3, 4), which was reported to be a significant economic burden on public health (5).

In some of the previous reports, epilepsy was shown to be associated with a higher risk of cardiovascular diseases (6-11). One study amongst adults with epilepsy showed that the prevalence of metabolic syndrome in sodium valproate and carbamazepine treated patients in Estonia was 20% and 40%, respectively (6). In addition, another study in India showed that 29.5% of young adults with epilepsy had metabolic syndrome 7. To the best of our knowledge, there are a limited number of studies regarding metabolic syndrome in children with seizure (10, 12). For instant, it has been reported that in Chinese children with obesity and epilepsy the prevalence of metabolic syndrome was 47.2%, which was not much more than the non-epileptic children with obesity (10). Another study among Indian children with epilepsy compared the effect of valproate vs. phenytoin monotherapy on lipid profile12. Due to the lack of sufficient data on the prevalence of metabolic syndrome in children with epilepsy, especially in Middle Eastern region, and conflicting results from previous pediatric studies sought us to evaluate the metabolic syndrome criteria and body composition in ambulatory children with epilepsy in comparison with age and gender matched healthy children.

## Material & Methods

**Study Design**

The present case-control study was performed in pediatric neurology clinics affiliated to Shiraz University of Medical Sciences, Iran April 2016 – April 2017. We included ambulatory children of 6–18 years old, having at least 2 unprovoked seizures more than 24 hours apart, and using sodium valproate or carbamazepine as anticonvulsanttherapy for at least one year. Children with a historyof paralysis and physical impairment, which have restricted normal activities and movements, and those with chronic disorders or endocrinopathies (e.g. liver disease, renal problem, familial dyslipidemia, diabetes mellitus) were excluded from the study. First, we enrolled 100 patients, but 19 children were excluded due to having at least one of the aforementioned exclusion criteria. Moreover, 4 parents / children refused to participate in this research, later. Ultimately, 77 children with epilepsy and 77 age / gender – matched healthy controls were participated in this study. The control group was recruited from local schools in Shiraz by considering their age and gender. An age/ gender – stratified randomly selection method was used to recruit the control group from ten government-dependent schools which distributed in four educational zone of Shiraz.

**Ethical considerations**

All the parents/guardians (both in case and control groups) signed a written informed consent. Local Ethics committee and vice-chancellor of research at Shiraz University of Medical Sciences approved 49 this research with grant number of 95 – 01 -49 –13302.

**Body Measurements, Body Mass Index (BMI), Pubertal Stage And Blood Pressure**

An expert pediatric endocrinologist evaluated the weight, height, waist circumference and pubertal stage of all participants. Weight was measured while the child was dressed a light clothing on a standard balance (Seca, Germany) and rounded to the nearest 0.1 kg. Height was measured while the child stood without shoes near a wall-mounted meter and rounded to the nearest 0.5 cm. Waist circumference was taken with a measuring tape at the midway level between the lateral rib margin and the iliac crest while the child stood upright and rounded to nearest 0.5 cm. BMI was also calculated by the following formula: BMI (kg/m2) = weight (kg) / [height (m)]2Puberty was evaluated according to the tanner standard system (13). Tanner stage 1 was considered as pre pubertal stage, tanner stages 2or 3 as early pubertal and tanner stage and 4 or 5 as late pubertal stages (13, 14). Arterial blood pressure was measured twice with 30 min interval through the standard auscultatory method using a sphygenomanometer, with suitable cuff size. The mean of two blood pressures was considered as the recorded blood pressure.

**Biochemical Laboratory Measurements**

Fasting blood samples were obtained to measure fasting blood sugar (FBS), cholesterol (Chol), Triglicerides (TG), high-density lipoprotein (HDL) and low density lipoproteins (LDL). Colorimetric method was used to measure FBS, TG, Chol, HDL and LDL using an auto analyzer (Biosystem SA, Barcelona, Spain).

**Body Composition**

Body composition was measured through a Hologic system dual – energy X-ray absorptiometry (DXA) (Discovery QDR, USA). The interpretation of body composition was done using the normative database of Hologic System DXA for children ages 5-23 (15). Fat mass index was also selected as a criteria for body fat composition (16).

**Definition of Metabolic Syndrome**

Metabolic syndrome was defined according to the latest International Diabetes Federation (IDF) consensus statement (17). Impaired glucose tolerance was defined as fasting glucose between 100–125 mg/dl; Hypertriglyceridemia defined as serum triglycerides higher than 90th percentile for age and gender specific range; low HDL-C was defined as HDL-C ≤ 10th percentile for age and gender specific range; hypertension was defined as blood pressure greater than 90th percentile for age, gender, and height specific range; and abdominal obesity was defined as waist circumference more than 90th percentile for age, gender and race specific range (17). Diagnosing the metabolic syndrome requires the presence of central obesity, plus any two of the other four factors.

**Statistical Analysis**

Statistical analysis was done using SPSS software, version 25. Data were indicated as mean ± SD and percentage. Student *t*-test and Mann-Whitney test were used to analyze quantitative data, and chisquare and fisher exact test were used to compare qualitative data between the two groups. Pearson correlation test was used to analyze two quantitative variables. Multiple regression test was used to evaluate the effect of some associated factors on metabolic, and body composition criteria. P-values 50 less than 0.05 were considered to be statistically significant.

## Results

This study included 77 epileptic children with average age 11.4 ± 3.2 years and 77 healthy age and gender-matched controls; half of each group were male. Prevalence of generalized tonic-clonic convulsions, partial seizure, and absence seizures were 71%, 16.9% and 2.6%, respectively. The most used anticonvulsant drugs were sodium valproate (72%) and carbamazepine (37%). Eighty percents of epilepsies were controlled with medications. In 79.2% of cases, seizures were controlled with one type anticonvulsant, 18.2% used two anti-conversant, and 2.6% used three or more anticonvulsants, simultaneously to control their seizures. All the descriptive data of patients and controls are shown in table 1. Serum FBS and TG of patients were more than the controls (P<0.001 and P<0.001). The fat mass index of patients was more than the controls (P = 0.012) and lean + BMC index in patients was lower than the controls (P = 0.017). There was no difference in age, gender, height-z score, weight-z score, BMI-Z score, systolic blood pressure, waist circumference percentile, serum HDL, cholesterol and pubertal stage of patients and controls (P>0.05). Prevalence of each metabolic syndrome criteria in both patients and control groups using either sodium valproate/carbamazepine or not are shown in table 2. Prevalence of metabolic syndrome in patients was more than the controls (7.8% *vs *1.1%, p-value = 0.032, 95% confidence interval: -0.127_ -0.005, table2 a ). Prevalence of hypertriglyceridemia, central obesity and BMI-Z score in patients was more than the controls with P = 0.009, P = 0.029 and P = 0.012, respectively (table 2a). Forward stepwise linear regression model was used to evaluate the association of multiple factors with each metabolic factor. Age, gender, height z-score, weight z-score, carbamazepine usage, sodium valproate usage, type of seizure, age of onset and pubertal stage were included in the analysis.

The analysis revealed that serum triglycerides in patients using carbamazepine was higher than non-using subjects (P = 0.019, Beta = 0.379, table 2 b). The data in table 2 b showed that waist circumference was more in high BMI-Z score (P = 0.026, Beta = 0.497) and higher ages (P<0.001, Beta = 0.445), blood pressure was also higher in older patients (P<0.001, Beta = 0.54), in higherheight z-scores (p = 0.038, P = 0.229) and in patients using carbamazepine for controlling their seizures (p = 0.016, Beta = -0.26). HDL-C wasmore in generalized seizures (p = 0.021, Beta =0.367). According to the fat mass index analysisin table 2 c, the data showed that it was lower in males (p = 0.013, Beta = -0.287), higher in higher BMI-Z scores (P = 0.006, Beta = 0.31), in patientsusing sodium valproate (p = 0.031, Beta = 0.238)and in late pubertal stage (p = 0.003, Beta = 0.35).

To further investigation, binary logistic Regression model was used to evaluate the associated factors (age, gender, height z score, weight z score, carbamazepine, and sodium valproate usage, type of seizure, age of onset, pubertal stage) with metabolic syndrome diagnosis. our findings showed that metabolic syndrome in children with seizure disorder was only associated with BMI z score (p = 0.038, Exp(B) = 2.3).51

**Table 1:** Descriptive data of patients and controls and the related comparisons

**Variable**		**Control group**	**Patients**	**P-Value**
Age (yr.)		11.4 ± 3.2	11.4 ± 3.2	0.945
Male gender (%)		50%	50%	----
Height (cm)		145.2 ± 15.9	143.2 ± 18.2	0.441
Height Z-score		-0.44 ± 0.99	-0.3 ± 1.3	0.421
Weight (kg)		36.9 ± 13.2	37.7 ± 15.7	0.703
Weight Z-score		-0.77 ± 1.1	-0.5 ± 1.5	0.175
BMI (kg/m^2^)		16.92 ± 2.3	18.1 ± 4.6	0.043
BMI Z-score		-0.66 ± 1.2	-0.74 ± 1.3	0.671
Waist circumference (cm)		65.3 ± 10.7	66.6 ± 10.7	0.447
Waist circumference percentile		33.9 ± 23.4	40.1 ± 26.8	0.115
Systolic Blood pressure (mmHg)		107 ± 11	10.3 ± 7.5	0.070
Blood pressure percentile		50.3 ± 29.9	47.4 ± 21.9	0.640
FBS (mg/dl)		75.2 ± 11.8	88.5 ± 7.1	<0.001
TG (mg/dl)		70 ± 62	105.7 ± 55.2	<0.001
HDL (mg/dl)		48.5 ± 22.2	44.5 ± 12.7	0.240
Cholesterol (mg/dl)		158.7 ± 29.7	163 ± 38.5	0.469
Fat mass index		4.05 ± 2.15	5.07 ± 2.21	0.012
Lean + BMC index		12.78 ± 1.98	11.8 ± 2.3	0.017
Puberty				
	Pre pubertal	44 (48.9%)	46 (51.1%)	0.800
	Mid pubertal	15 (16.7%)	17 (18.9%)	
	Late pubertal	31 (34.4%)	30 (46.6%)	

**Table 2a, b and c:** Metabolic syndrome criteria in patients and controls and the related comparisons: a. information of total patients vs. controls; b. information about patients using carbamazepine or not; c. information about patients using sodium valproate or not.

**2a.**						
**Criteria**		**Controls**		**Patients**	**P-Value**	
Hypertriglyceridemia (%)		14.4%		31.2%	0.009	
FBS ≥ 100 mg/dl		2.2%		5.2%	0.304	
Low HDL		38.9%		27.3%	0.113	
Central obesity		0%		5.2%	0.029	
Hypertension		3.3%		3.9%	0.846	
Metabolic syndrome		1.1%		7.8%	0.032	
BMI Z-score ≥ 2		0%		6.8%	0.012	
**2b.**						
**Criteria**	**Patients using carbamazepine**		**Patients did not use carbamazepine**		**P-Value**	
Hypertriglyceridemia (%)	34.5%		24.5%		0.327	
FBS ≥ 100 mg/dl	6.9%		3.3%		0.436	
Low HDL	31%		24.6%		0.518	
Central obesity	0%		6.6%		0.158	
Hypertension	3.4%		3.3%		0.967	
Metabolic syndrome	6.9%		6.6%		0.952	
BMI Z-score ≥ 2	7.1%		5.2%		0.714	
**2c.**						
**Criteria**	**Patients using sodium valproate**		**Patients did not use sodium valproate**			**P-Value**
Hypertrigliceridemia (%)	32.1%		20.6%			0.235
FBS ≥ 100 mg/dl	3.6%		5.9%			0.606
Low HDL	25%		29.4%			0.646
Central obesity	7.1%		0%			0.111
Hypertension	3.6%		2.9%			0.872
Metabolic syndrome	7.1%		5.9%			0.816
BMI Z-score ≥ 2	7.4%		3.1%			0.412

## Discussion

The present study showed that the prevalence of metabolic syndrome in children with seizure disorder was more than the healthy children (7.8% vs 1.1%, p-value = 0.032, 95% confidence interval: -0.127_ -0.005). In addition, we showed that fat mass index in children with epilepsy was higher than the healthy children who did not affected by epilepsy; However, lean + Bone mineral content in children with epilepsy was lower than their healthy controls. Amongst metabolic criteria, hypertriglyceridemia and central obesity were higher in children with epilepsy. In addition, we revealed that carbamazepine usage was associated with higher TG and higher blood pressure, but sodium valproate usage was linked to a higher fat mass index. Previous reports in adults showed a high prevalence of metabolic syndrome among patients with epilepsy (7), ranging from 29.5% in Indian young adults to 20% in Estonia (6) as well as 43% in overweight patients with epilepsy in Italy (8). Difference in the prevalence might be due to patient’s selection criteria, such as age, weight, anticonvulsants medications, and race (6- 8). Limited number of studies have been performed regarding the prevalence of metabolic syndrome in children with epilepsy. They showed a relative higher rate of hyperlipidemia in Indian children using sodium valproate, (12) and 47% prevalence of metabolic syndrome in Chinese patients with epilepsy and obesity using sodium valproate (10).

As far as we know, the present study was the first to have evaluated a relatively large number (77) of ambulatory children with epilepsy compared to healthy controls. The present study also showed that 7.8% of the children had IDF criteria of metabolic syndrome, which was higher than healthy children. In patients with epilepsy, a sedentary lifestyle and anti-epileptic drugs (AEDS) can lead to obesity and metabolic syndrome (18, 19). Some anticonvulsants, such as sodium valproate, phenytoin, and carbamazepine, increase the risk of hyperlipidemia, obesity, and metabolic syndrome (6, 8, 9, 11, 20). Verrotti et al., showed that adult patients who gain weight during sodium valproate therapy could develop metabolic syndrome and cardiovascular disease (8), and females could be at higher risk (6), but it is independent of age, seizure type, and duration of seizure (10). The present study showed that carbamazepine usage was associated with higher serum TG level and higher blood pressure. Moses et al., showed that carbamazepine was associated with abnormal lipid profile and high total cholesterol 20. This might be due to the effects of carbamazepine on cytochrome P450 enzymeinduction, resulting in abnormal lipid level (12).

Ciszowski et al., revealed that carbamazepine could affect different cardiovascular parameters, such as blood pressure in toxic doses 21. Carbamazepine could have an effect on cytochrome p450 and could cause a marked decrease in serum concentrations and efficacies of CYP3A and / or p-gp substrates, resulting in blood pressure changes (22). The present study showed that children with epilepsy had a higher fat mass index and lower lean + BMC index. In addition, we revealed an association between using sodium valproate and higher fat mass index in children with epilepsy. In line with our report, Rauchenzauner et al., showed that sodium valproate was associated with an increase in weight, body fat, and serum leptin in children with epilepsy (23), and lower bone mineral content (14). Effect of sodium valproate on body fat mass could be explained by these mechanisms. It could dysregulate the hypothalamic system, and affect serum adipokines, hyperinsulinemia and insulin 54 resistant (11). Effect on hypothalamic system might be explained by valproate effects on the gammaaminobutyric acid (GABA) transmission

enhancement in the hypothalamic axis (24, 25). In addition, sodium valproate could increase the expression of adipokines in the brain and pituitary (cephalokines), which regulates resistin and angiopoietin-like protein 4 (ANGPTL4) that might have a role in obesity and insulin resistance (26). On the other hand, sodium valproate might directly affect adipose tissues’ adipokines, such as adiponectin, leptin, and ghrelin, contributing to changes in fatty acid oxidation, weight gain, enhancing patient’s appetite, influencing glucose and insulin metabolism that leads to obesity, and higher body fat proportion (27-30). The present study has several strong points. As we know, this is the first case-control study that has evaluated metabolic syndrome amongst children with epilepsy in the Middle East. In addition, this is the first study in the Middle East that has evaluated the body composition indices in addition to metabolic criteria in children with epilepsy by considering the confounding factors, such as weight-z score, height z-score, pubertal stage and ambulation, to have a better estimation of the metabolic factors prevalence and associations. Despite these strong points, we had some limitations. It is better to investigate the insulin level for calculating the Homeostatic Model Assessment of Insulin Resistance (HOMA) index, which we have considered in our future works. Second, it is better to perform this study via a prospective method to compare the metabolic syndrome during the course of therapy.

**In Conclusion**, Our study revealed that children with epilepsy are prone to metabolic syndrome and higher body fat mass. Hence, early diagnosis and prevention of metabolic syndrome criteria in patients with epilepsy, performing regular exercise, and having a healthy diet should be encouraged during their childhood.
